# Sense of safety or meaning in danger? Real-contact stick fighting as an imagistic ritual

**DOI:** 10.3389/fpsyg.2024.1327396

**Published:** 2024-07-02

**Authors:** Teemu Pauha

**Affiliations:** Faculty of Theology, University of Helsinki, Helsinki, Finland

**Keywords:** martial arts, Dog Brothers, ritual, extreme sport, edgework, identity fusion, initiation, masculinity

## Abstract

It is a common assumption that human behavior is guided by a desire to feel safe and avoid harm. However, this view is challenged by the popularity of high-risk leisure sport and other practices that involve subjecting oneself to a considerable danger with no apparent gain. By using real-contact stick fighting as an example, I suggest that the attractiveness of at least some such practices can be explained by cognitive dynamics that are typical of affectively intense rituals such as initiations. Affectively intense rituals are known to enhance personal meaning-making and foster identity fusion, that is, the overlapping of personal and social identities. The sense of meaning thus engendered effectively satisfies common identity motives and thus elicits positive affect. By introducing ritual studies perspectives into the edgework paradigm that is commonly used to conceptualize voluntary risk taking, I contribute to an increased understanding of the cognitive processes motivating participation in extreme leisure sport.

## 1 Introduction

*When they talk about stickfighting being a male-initiation rite in the traditional sense, a truly transforming experience, it’s not some New Age con* (Jacobs, 1998).

Psychologists and other behavioral scientists often assume that people are naturally disposed to maximizing their safety and avoiding danger (Willig, 2008, p. 691). If this is the case, why do people voluntarily put themselves in harm’s way and engage in behaviors that pose a significant threat to their health and even life? In particular, why would anyone willingly put themselves in danger when they could accomplish the same thing with no risk to their well-being? For example, what attracts people to climbing steep rocks “free-solo”—that is, without ropes and harnesses—when they could get on top of the same rock quite safely by having someone belaying them? It seems that, at least for a segment of the population, risk of death and injury has intrinsic value that increases the attractiveness of a leisure activity.

In this paper, I discuss another phenomenon that involves a conscious decision not to use protective gear that would make a potentially deadly activity relatively safe. The Dog Brothers are a group of martial artists focusing especially on armed combat with strong FMA (Filipino martial arts) influences. “Filipino martial arts” (FMA) is an umbrella term referring to a group of martial arts also known as kali, escrima/eskrima, or arnis (Parkes et al., 2023, p. 18). In the Western context, the most widely known and practiced forms of FMA include such systems as Balintawak Eskrima, Doce Pares Eskrima, Kombatan, and Pekiti Tirsia Kali. In the world of martial arts, FMA is distinguished by its strong emphasis on armed combat. A wide variety of weapons are used in the context of FMA, but the most common of these are sticks and blades of varying length.

Martial arts and even armed combat can be practiced safely by wearing a helmet and other protective equipment, fighting with fake or padded weapons, etc. However, among martial artists, the Dog Brothers have become famous for eschewing such safety measures and fighting with sticks and other melee weapons with almost no protection and practically no rules. The mission statement on the website of the Dog Brothers Canada (n.d.) expresses the essential: “[f]ull power, minimal equipment, anything goes, stick fighting.” By consciously disregarding common safety precautions, the Dog Brothers expose themselves to serious injury or death. This article is an attempt to figure out why.

## 2 Starting points

Lyng’s (1990) concept of “edgework” has become extremely influential in the study of conscious risk-taking. According to Lyng (2004a, p. 5), it is easy to explain why people put their lives into danger without a concrete gain. In his view, people do so because of being seduced by the thrill—or in other words, because “it’s fun!” What is more difficult for Lyng (2004a, p. 5) to explain is how certain life-threatening activities acquire such a seductive character.

In what follows, I provide my own response to Lyng’s call and try to understand what makes the Dog Brothers style of fighting so attractive. Drawing on ritual studies, and in particular the cognitive study of religion (CSR), I argue that the Dog Brothers “Gatherings of the Pack” serve as initiation rites that provide their participants with a sense of identity. What makes this identity distinctive is the way in which it fuses personal and social aspects of the self. Put differently, it is by being joined in the community that the Dog Brothers become authentic individuals. This new self is symbolized by being given a new name that is at the same time uniquely individual and deeply shared.

I illustrate my argument with examples from official and unofficial communications of individuals and groups associated with the Dog Brothers. These include, for example, websites of national associations, blog posts, and media interviews. Somewhere in between official and unofficial are the writings and interview comments of Marc “Crafty Dog” Denny, who is one of the original Dog Brothers and has assumed the position of a *de facto* spokesperson for the group. Denny is by far the most active representative of the Dog Brothers to the outside world and, consequently, many of the following examples are from him.

I suggest that the accounts of first-time participants at the Gathering may be particularly fruitful for understanding the attractiveness and psychological effects of the Dog Brothers style of fighting. Having joined the Dog Brothers fold fairly recently, such participants are perhaps in the best position to remember their reasons for doing so. Furthermore, while full-contact fighting often involves fear and aversion, such emotional reactions tend to decrease with practice (Vaccaro et al., 2011, pp. 431–432). The more one fights, the more one becomes habituated to both inflicting and experiencing pain (Thornton et al., 2017). Therefore, any effects pertaining to emotional and physiological arousal are likely to be pronounced in the accounts of the first fights. Because the ritual dynamics in which I am interested depend on arousal, I have systematically searched the internet for personal accounts of participating in a Dog Brothers Gathering for the first time. The accounts were searched on Google using search terms such as “Dog Brothers” and “first Gathering” or “first fight.” I ignored search results that did not describe personal experiences of fighting but, for example, provided general advice for someone preparing for their first Gathering. The final dataset included five accounts of varying lengths, which had been published in personal blogs, newspaper interviews, or discussion forums.

Despite utilizing examples drawn from the field, I have not engaged in in-depth data analysis. The examples are intended to illustrate a theoretical argument and not to demonstrate an empirical finding. Instead of presenting a detailed analysis, I propose what I believe is a fruitful theoretical synthesis, the testing of which remains for a future study.

Furthermore, despite using the Dog Brothers Gathering of the Pack as an example, I argue that the notion of imagistic rituals has a more general applicability in the context of extreme leisure sports. Accordingly, towards the end of my article, I discuss whether the dynamics that I have described apply also to individual sports, such as rock climbing. In addition, I reflect on the factors that make the identities produced through imagistic rituals psychologically appealing. Finally, I conclude the article by outlining suggestions for future research.

In addition to my academic pursuits, I am a long-time martial artist with experience in both Filipino and other martial arts. I have participated in seminars taught by the Dog Brothers, but I have never fought in an actual Gathering of the Pack. Therefore, when analyzing the ritual dynamics of the Gathering, I cannot draw on any personal experience of it. However, I do have enough experience in martial arts to understand their general appeal, including that of high-intensity sparring. I can empathize with the decision to fight at the Gathering and can easily access the part of me that could make the same decision.

### 2.1 Safety and security in psychology

An underlying if often implicit assumption in much of behavioral research is that people are naturally prone to maximizing their safety and avoiding risk. Most famously, Maslow (1943) identified safety as the second most important basic human need, following immediate physiological needs like water and food. Likewise, Murray (2008, pp. 124 and 144) included “harmavoidance” in his list of manifest needs and considered it to have a biological basis in the human nervous system.

In addition to needs, safety and security have also been perceived as basic values (Schwartz, 1994) and motives (e.g., Pincus, 2024, p. 734). Writing from a psychoanalytic perspective, Greenberg (1991, pp. 132–133) refers to safety as both a drive and a motive, even arguing that “the importance of feelings of safety is one of the strongest findings that has emerged from a century of psychoanalytic investigation.”

According to Gilbert (2020), human behavior is guided by two motivational tendencies: threat vigilance and resource seeking. These two tendencies work in opposition to each other, in that the perception of threat triggers avoidance behavior. In contrast, in the absence of threat, an individual experiences a sense of safety, which encourages the exploration of one’s surroundings.

Studies into human decision-making and economic behavior often take the human tendency of risk avoidance for granted—so much so that Zhang et al. (2014) refer to risk avoidance as “one of the most fundamental properties of human behavior.” Assuming that human behavior is guided by the maximization of personal safety makes evolutionary sense. After all, needlessly subjecting oneself to the risk of death or injury would decrease one’s possibilities of transmitting one’s genes to future generations. In this kind of framework, it is difficult to understand what could motivate people to consciously risk their lives—especially when there are no apparent gains in doing so.

### 2.2 Edgework paradigm and its limitations

Willig (2008) has criticized psychological scholarship for wrongly assuming that people are naturally disposed to maximizing their well-being and therefore perceiving conscious risk-taking as a symptom of psychopathology. Instead of being irrational, conscious risk-taking may represent an alternative rationality that does not acknowledge the ultimate value of safety or well-being. Qualitatively oriented sociologists and psychologists have attempted to unpack such alternative rationalities and the ways in which risky behavior may play an important role in personal meaning-making (for an overview, see Willig, 2008, pp. 692–693). In this regard, an especially important innovation is the concept of edgework, originally conceived by Lyng (1990).

For Lyng (1990, pp. 856–858), “edgework” is an umbrella term for activities ranging from drug use to combat soldiering and from extreme sport to criminality. What is common to all these is that they involve negotiation of some ultimate boundary, be it the boundary of sanity and insanity, of order and disorder, or of life and death (Lyng, 1990, p. 858, 2004a, p. 4). As the term suggests, edgeworkers take it to the very limit, trusting that their skills will prevent them from slipping over it.

In his original formulation of the edgework concept, Lyng (1990) combined Marxist and Meadian perspectives to argue that extreme sports and other edgework activities serve as an antidote to the routinization that characterizes the capitalist society. To give a rough summary of his detailed argument, edgework provides opportunities “for acquiring and using finely honed skills and experiencing intense sensations of self-determination and control, thus providing an escape from the structural conditions supporting alienation and over-socialization” (Lyng, 2004a, p. 5). This modernist conception of edgework has since then been challenged—or perhaps supplemented—by postmodernist views. In a postmodern perspective, edgework does not provide an escape from an alienated to a more authentic existence. In the postmodern condition ruled by “simulations of simulations” (Lyng, 2004b, p. 37), there is no one identity or way of living that is more authentic than others. The most that edgework can offer is an identity that is different to the ones associated with work and other spheres of life.

Lyng is not alone in presenting edgework as a product of modernity. In fact, the standard, if only implicit, assumption in much of sociological research is that extreme sport is inextricably bound to the modern condition (see, for example, Poulson, 2016; Kidder, 2017, p. 103; Simon, 2019)—that the contemporary society entails some essential social pathology that predisposes a segment of the population to put themselves in unnecessary danger. However, neither extreme sport nor voluntary risk taking more generally is a modern phenomenon. There is archeological evidence suggesting that ancient Minoans practiced “bull-leaping,” the goal of which was to grasp the horns and vault onto the back of a charging bull (Mcinerney, 2011, pp. 8–9). A more well-known ancient example is pankration. Similarly to modern MMA, pankration combined stand-up fighting (punching, kicking, and wrestling) and grappling on the ground (Gardiner, 2020, pp. 310–311). The only banned techniques were biting and eye gouging. Pankration was fought bare-handed, but historical examples of armed combat sports are also plenty. For example, in 17^th^ century Venice, thousands of common workers participated in “battagliola,” that involved two sides fighting with pointed sticks and other weapons over the control of a bridge (Davis, 1994).

By linking edgework to social conditions of modernity, Lyng and his kin fail to explain the attraction that it has held in other times. In this article, I suggest that at least some forms of edgework enjoy universal appeal because they tap into common processes of social cognition. Indeed, and as I will discuss in more detail in the next sections, the Dog Brothers Gathering of the Pack resembles the initiation rites practiced in many preindustrial societies in that it engenders intense arousal that fosters the experience of communitas.

## 3 Gathering of the pack from a ritual studies perspective

### 3.1 Dog Brothers gathering of the pack

The Dog Brothers are a self-identified group of “sweaty, smelly, psychopaths with sticks” (dogbrothers.ch, 2012). The group was established in California in 1988 by Marc Denny, Eric Knaus, and other FMA practitioners who had started to explore stick fighting with minimal protection. Fittingly to my purpose of applying the cognitive science of religion in the context of martial arts, the Dog Brothers have their own origin story, or a foundational myth (Wilson, 2009; dogbrothers.ch, 2012). According to the story, the original group engaged in three days of continuous fighting—an event that has become known as the “Rumble at Ramblas” (RAR). This experience “forged a special bond” (Wilson, 2009) among the participants and gave them a deep sense of comradeship (dogbrothers.ch, 2012). In order to formalize this bond, the participants adopted nicknames that signified their membership in a special community. Inspired by a Conan the Barbarian comic, the group started to call itself the “Dog Brothers” and its founding members became known as “Crafty Dog” (Denny), “Top Dog” (Knaus), etc.

The Dog Brothers distinguish themselves among other FMA groups by practicing what they call “real-contact stick fighting” (RCSF). The choice of term is a conscious attempt at demarcation from more widely known “full contact” matches (Wilson, 2009). Unlike full contact fights that typically involve protective padding and rules that forbid the most dangerous techniques, RCSF is characterized by the bare minimum of safety precautions. RCSF matches are fought with no holds barred and the only protective gear is a fencing mask and street hockey gloves (Wilson, 2009). Their purpose is to protect the eyes and the finger bones, the latter of which are almost certain to receive heavy blows in a stick fight and, if broken, would stop all martial arts practice for a long time. Having as little protective gear as possible is an explicit goal. The original Dog Brothers have even lamented in interviews that modern fencing masks provide too much protection: it has become more difficult to knock someone out in a match (Wilson, 2009).

My aim in this article is to argue that real-contact stick fighting is a heavily ritualized activity. This is already apparent from its occurrence during specific and, one could argue, highly symbolic times of the year. The formal setting for the real-contact stick fighting is the Gathering of the Pack. In the United States, Gatherings are organized twice a year: on the first Saturday of May and on the Saturday of September closest to the autumnal equinox. The Canadian and European Dog Brothers have their own Gatherings. In addition to the Dog Brothers themselves, Gatherings are open to other martial artists who wish to test their skills in a “real-contact” match. In fact, in order to be accepted into “the Dog Brothers Tribe” in the first place, one needs to have attended and fought in Gatherings.

In some sports, a championship title or a prestigious prize are major factors that motivate participation. In contrast, the fights at the Gathering are fought without a referee. Consequently, there are no declared winners and no trophies. However, instead of a championship title, there are other titles to be earned. If a fighter is not yet a Dog Brother but demonstrates “good spirit” and “fighting ability” to the Dog Brothers in attendance, she or he may be granted entry into “the tribe” and assigned a new name to signify this (DBMA, 2023b). Every new member shares the same name, “Dog,” which also emphasizes their common identity. Further Gatherings provide opportunities for ascending further in the tribal hierarchy. Eventually, one may reach the level of a full Dog Brother. At this stage, one receives a unique Dog Brothers name that is intended to reflect one’s own style of fighting or personality (e.g., “Lucky Dog,” “Tricky Dog”) (Conder, 2011).

### 3.2 Initiation into and within the tribe

Being transformed by passing through ordeals and receiving a new name to signify this transformation are key components of initiation (*initium*, lat. “beginning”) (Eliade, 2005, pp. 4475–4476). Initiation is one, and arguably the most prototypical, example of rites of passage (Grimes, 2013, p. 202). According to the foundational work on rites of passage (Van Gennep, 1960, p. 26), a society resembles a house with many rooms. During their life course, a person passes from one room to the next, or from one social status to another. Similarly to moving between rooms, moving between statuses involves the crossing of a threshold. Van Gennep (1960, pp. 20–21) refers to this threshold as “the liminal” (*limen*, lat. “threshold”). The liminal is a space between socially recognized statuses, a space of not-anymore and not-yet (Turner, 1977, p. 95). Being free from the roles and hierarchies that normally divide them, people in the liminal space can experience exceptional togetherness, or *communitas* (Turner, 1977, p. 96).

A fight at the Gathering marks a liminal space where the rules and roles of everyday life no longer apply. The fighters are no longer separated by education, profession, or class. In a fight, such markers of social boundaries disappear, and the relationship between the fighters is defined only by their mutual goal to strike each other with a stick or other weapon. A fight, to quote Turner’s (1977, p. 96) description of the liminal, is ‘a “moment in and out of time,” and in and out of secular social structure, which reveals, however fleetingly, some recognition (in symbol if not always in language) of a generalized social bond that has ceased to be and has simultaneously yet to be fragmented into a multiplicity of structural ties’.

The temporary dissolution of social divisions that occurs during a fight bears a marked resemblance to the communitas that characterizes the liminal phase. Accordingly, despite the violence inherent to it, the relationship between the fighters is not antagonistic, and RCSF is closer to mutual play than competition-oriented sport. This is also reflected in the one and only rule that the fighters are expected to follow at the Gathering: “Be friends at the end of the day” (Denny, 2023). A spirit of non-competition is also prevalent in Richard Killick’s recollections of his first Gathering (Taylor, 2009):

*The idea is to build fighters, not to break them, so there’s a lot of respect amongst the fighters and a feeling of comradeship throughout. You get applause just for getting up for a match. As far as picking fights are concerned it’s a bit like asking someone to dance at a club, except you’re asking them if they would like to fight!*

The same ethos is manifest on the Dog Brothers website, where Denny (2023) reflects on the proper attitude to have when fighting at a Gathering:

*The spirit of the fights is that of members of the same tribe helping each other to prepare to defend the land, women, and children of the tribe. Both going too hard and going too soft are counterproductive. In this spirit, what might be too much for one man to handle, could be too little for another. It is a sign of respect for your “opponent” to really go after him-you are saying you respect and believe in his skill and spirit to deal with it, yet at the same time even in the adrenaline of the moment you are looking out for his welfare so as to not damage him and thus weaken the tribe. It is in your best interest that he be as good a warrior as possible when you stand together in battle.*

As expressed by the quotation marks around the word “opponent,” the two people participating in the same fight at a Gathering are not fighting to defeat and subdue each other but to push each other to a higher level. In the very next paragraph, Denny (2023) makes it explicit that this higher level is characterized not only or even primarily by physical superiority but the goal of RCSF is a more general transformation as a human being:

*A stickfight is an intense adrenal experience. The Learning that takes place in this altered state is of an entirely different order from ordinary learning. The greater the intensity of the fight, the greater the need to simultaneously tap into a centered awareness that keeps you from taking the shot that would be too much. The cultivation of this duality, i.e. greater adrenaline & greater centering, is what we mean by the full credo: “The greater the dichotomy, the profounder the transformation. Higher Consciousness through Harder Contact.” It is our hope and belief that this deeper learning carries over to the rest of one’s life; and should one ever need to use one’s skills that it will be done with a calmness that allows for good judgement as well as good skill.*

Grimes (2013, p. 202) has drawn a sharp distinction between “a rite of passage” and “a ceremony.” According to him, a true ritual entails a transformation of the participant. If an activity merely confirms a previously occurred transformation, it is not a ritual but a ceremony. As the excerpt above makes clear, the vocabulary of fundamental transformation is central to how the RCSF is described as an experience. What looks like a struggle of two people against each other is in fact a joint struggle of two people to break through to the next level. The ideology behind this is succinctly expressed in the oft-recited creed “Higher Consciousness through Harder Contact.” Furthermore, certain passages portray the transformation as not incremental but total: “it is probably a good thing to have a phase in one’s evolution where one does [RCSF] a lot so as to step through a certain door” (Denny, 2023). The metaphor of stepping through a door suggests that with intense practice, it is possible to leave one state of being behind and enter into a different existence. This new existence is characterized by a new kind of consciousness: “We come to see our fears as no different that [*sic*] a dog’s fear of a vacuum cleaner” (Denny, 2023).

Being written by the head instructor of the Dog Brothers Martial Arts, the excerpts quoted in the previous paragraphs could be discounted as mere marketing rhetoric. However, the same themes of community and transformation are also central to Richard Killick’s recollections of fighting at the Gathering for the first time:

*Paul Taylor: Eric Knaus [“Top Dog”, one of the original Dog Brothers] has said that once you experience a fight like this (a hard, full contact stick fight) it changes you. Would you agree with this?*

*Richard Killick: Absolutely! It also highlights your weaknesses straight away. You have to become a total fighter rather than just someone who fights at a specific range.*

*Paul Taylor: No spiritual enlightenment then?*

*Richard Killick: No, but then I’ve been under enemy fire while I was in the Army and have been through the fear thing before. I would say though that as in all hard fights it makes you close to your opponent. There’s a feeling at the end of it that you’ve been through something together. So there’s that comradeship and it’s stressed a lot because at the end of the day your opponent is helping you to grow.*

In this section, I have argued that the Dog Brothers Gathering of the Pack can be understood as a rite of passage. I have drawn especially on the work of Van Gennep (1960) and Turner (1977), who pioneered the study of rites of passage and analyzed in detail the common characteristics of rituals used to navigate the transition from one social status to another. Their work has since then been supplemented by various cognitive theories of ritual. In the next section, I introduce an especially important cognitive account of religion, Harvey Whitehouse’s theory of divergent modes of religiosity.

### 3.3 Doctrinal and imagistic modes of martial arts

Study of religion has long recognized a distinction between two broad types of religiosity (Whitehouse, 2002, pp. 293–294). In recent scholarship, these two have been referred to as “lived,” “everyday,” or “popular” religion, on one hand, and as “institutional” or “official” religion, on the other (see, for example, Ammerman, 2016). To provide a cognitive account of this common duality in religion, Whitehouse has formulated the theory of divergent modes of religiosity (DMR). According to the DMR theory, the two broad types, or modes, constitute different methods of religious transmission (Whitehouse, 2002). Whitehouse referred to the two modes as “doctrinal” and “imagistic,” respectively.

The “doctrinal mode” relies on the processes of semantic memory. In order to be efficiently encoded in the semantic memory, information needs to be frequently repeated, for example, in recurring rituals such as Islamic *salat* (prayer). Religious knowledge that is transmitted in the doctrinal mode tends to bear the characteristics that are typical for information stored in the semantic memory: as the term “doctrinal mode” suggests, it tends to consist of official doctrines or other general facts about the religious tradition. Such knowledge is often easy to verbalize, but it is usually difficult to recall exactly where and how the knowledge was originally acquired.

Transmission of martial arts expertise typically occurs in what Whitehouse (2002) refers to as the doctrinal mode. That is, the various techniques are practiced and rehearsed on a regular basis, with the goal of assimilating the correct body mechanics. The exercise is typically more or less choreographed, with each participant carrying out a set of predetermined movements in a predetermined sequence. This is also the usual way in which FMA is trained. In a typical case, FMA training involves two persons, one of whom (“the feeder”) makes an attack that the other one (“the responder”) counters (Parkes et al., 2023, p. 24). Usually, a FMA system includes a specific set of attacks, each of which have their own specific counters.

Martial arts skills attained in the doctrinal mode are stored in implicit memory. That is, one can perform a technique such as a punch, block, or kick and often also verbally describe it, but one typically cannot recall the conditions in which one first learned the technique. Furthermore, many common FMA practices are designed to facilitate efficient encoding in the implicit memory. An example are the flow drills that have become something like a trademark of FMA. A flow drill consists of a continuous stream of attack and defense in which the roles of the feeder and responder constantly alternate (Parkes et al., 2023, p. 24). The goal of the drill is to condition the practitioner to automatically react to a certain attack with a certain response.

In contrast to the doctrinal mode and its reliance on semantic memory, the “imagistic mode” is based on the processes of the episodic memory system. The information that is stored in episodic memory is often experienced as being deeply personal. Such information is strongly associated with specific events in the autobiography and consequently, it cannot be rehearsed on a regular basis. Imagistic rituals are by definition rarely performed (Whitehouse, 2002, p. 303). Similarly, real-contact stick fighting is a relatively infrequent occurrence even in the lives of active martial arts practitioners. The Dog Brothers in the US hold two public Gatherings a year, in addition to which there may be some closed Gatherings. Instead of frequent repetition, episodic memory encoding relies on emotional significance. Events that evoke strong emotions are likely to be of personal relevance and therefore worth keeping in mind. Rites of transition, and especially those that involve strong physical or emotional sensations (such as pain or fear), are textbook examples of religion in the imagistic mode. In a similar vein, and in response to the question “Do you guys do this all the time?,” the Dog Brothers FAQ states: “To do this all the time is something we have not done since we were much younger” (Denny, 2023). The FAQ continues by referencing the three days of continuous fighting at the “Rumble at Ramblas.” For the participants, the Rumble was a “transformational experience” that does not need to be repeated on a regular basis.

Second key characteristic of an imagistic ritual is a high level of emotional or physiological arousal (Whitehouse, 2002, p. 303). Reminiscing his very first fight at a Gathering, Krischel (2018) provides an avid description of the adrenaline rush that it involved: “It’s a blur. I mean, sparring is usually a blur anyway [—], but this is really a blur. Not enough time to think — it is reflex upon reflex upon reflex. [—] Footwork happens. Strikes happen. Breathe. Move. Hit. Things start to flow. I’ve given up thinking, and hits are landing.”

Judging by its infrequency and emotional intensity, the Dog Brothers Gathering of the Pack, like most initiations, bears definite characteristics of imagistic rituals. This pronounced imagistic character, in turn, has consequences for the formation of the Dog Brothers as a community.

### 3.4 Rituals bind people together

Studies have found that doctrinal and imagistic modes serve different kinds of identity formation. Doctrinal rituals such as sermons provide believers with factual information on what they should do and think as members of a certain religious group. Thus, in the terminology of social identity theory, doctrinal rituals produce social identity content (on social identity content, see Evans et al., 2023).

In the context of social identity theory, it is customary to distinguish social identity (a person’s sense of group belonging) from personal identity (a person’s sense of individuality) (see, for example, Onorato and Turner, 2004, p. 259). Classical social identity theory suggested that the two kinds of identity constitute opposite ends of a continuum in that personal identity activation reduces social identity activation and vice versa (see, for example, Tajfel, 1982, p. 13). In other words, in social interaction, a person may perceive themselves and others either as distinct individuals or as representatives of a group. Classic examples are marriage and war, respectively: Typically, for a married person, their partner is first and foremost an individual, not a representative of a group. In contrast, soldiers do not wage wars because of interpersonal animosity against the individual soldiers on the opposing side, but because the soldiers on the opposing side are perceived as belonging to a group that is hostile to one’s own group. The same person may be “Sid” or “Nancy” in the context of marriage, but “Ukrainian” or “Russian” in the context of war.

However, more recent research has identified a special condition of identity fusion in which the antagonism between personal and social identities does not hold (Swann et al., 2009). Instead, in identity fusion, the personal and social identities become merged so that a person experiences the membership in a group as the essence of their most authentic individuality. If a person were completely fused with their nation, for example, Ukraine, they would be first and foremost “Ukrainian” even in the context of marriage, because their perception of their own individuality is defined by their nationality.

Studies have shown that while doctrinal rituals provide social identity content and thus promote self-categorization into a group, imagistic rituals foster identity fusion (Whitehouse, 2018, 2021, pp. 93–99). Imagistic rituals such as initiations are life-defining. Their sheer emotional intensity ensures that they leave a lasting mark on one’s autobiography and therefore also one’s personal identity. If there are others who have participated in the same ritual, they are perceived as having been similarly affected. They have been through the same ordeal and emerged similarly transformed, with the same “autobiographical essence” (Whitehouse, 2018, p. 4). In other words, after being involved in a shared ritual, a person perceives that the same experience defines both their own being and the whole group of ritual participants.

In Section 3.2, I quoted Richard Killick’s recollections of fighting at the Gathering for the first time. In his recollections, Killick notes how those fighting on the opposite sides of the same combat are bound together by invisible ties. The effects described by Killick can be explained with identity fusion: violence leaves its mark in the life story of everyone involved, and this shared “autobiographical essence” (Whitehouse, 2018, p. 4) both binds them together and sets them apart from everyone else.

The group-bonding effects of a ritual increase with the intensity of the arousal (Whitehouse, 2021, p. 84). Accordingly, pain and fear are key elements of imagistic rituals all around the world. As can be expected, pain, as well as fear of pain and injury, are also natural accompaniments of RCSF. Krischel (2018) recollects his two first impressions of his first opponent at a Gathering: “Nice guy from Minnesota. He’s got an aluminum balisong [= butterfly knife] trainer that looks like with enough force, it could turn into grievous puncture wound in any number of sensitive spots.” To provide a further example, “Stickgrappler’s” (2018) report of his first Gathering is essentially a succinct list of hits received:

*Got hit in my head with his Caveman (diagonal slash down) from his Dominant/right hand. The first ever head shot where my head felt the hit! All other head shots I ever got I can hear I got hit but the mask took the hit and my head didn’t feel those … he hit me in my left elbow where I had bursitis a few years ago. Swollen now. Hit me on the ribs with nasty looking stick hickey that hurt a little but is nothing.*

So far, I have concentrated on the individual cognitive dynamics that are involved when participating in imagistic rituals. However, it is worth noting that the difference between the two modes characterizes also the social formation of different martial arts groups (cf. Whitehouse, 2002, p. 309). Similarly to religious communities in which the transmission of tradition occurs predominantly in the doctrinal mode, a typical martial arts club is open to everyone interested and often actively seeking to attract new members. The authority in the club in centralized, in that there is a single head coach or “master” who determines how each technique should be correctly executed. Consequently, uniformity of style and technique is enforced in the club.

Compared to this, the social formation of the Dog Brothers is rather different and resembles that typical of religious communities that emphasize the imagistic mode in their practice (cf. Whitehouse, 2002, p. 309). Even though the Gatherings are open to everyone, the Dog Brothers Tribe itself is markedly exclusive, with entry being granted only to those who demonstrate both the desired character and martial prowess. The leadership in the Tribe is diffuse, and all Dog Brothers have a say, for example, on ascensions in the Tribe hierarchy. The Dog Brothers have no set curriculum that would define in detail the correct “Dog Brothers style of fighting.” Indeed, individual Dog Brothers often have their own styles that they practice and teach. For example, Burton “Lucky Dog” Richardson has his own “JKD Unlimited” martial arts system. Instead of a shared curriculum or centralized community structure, the cohesion among the Dog Brothers is maintained by intense affective bonds. As the accounts of those who have fought at a Gathering demonstrate, real-contact stick fighting is often experienced as a transformational event that leads to a strong sense of community among those involved.

### 3.5 Gendered dynamics of the gathering

Before concluding, I would like to comment on the gendered dynamics of the Gathering of the Pack. As the name “Dog Brothers” suggests, there is a general assumption of the fighters being male. The assumption is conveyed in many ways in the official and unofficial communications of the group, but it is also reflected in the gender distribution at the Gatherings. For example, in an interview for the *Black Belt* magazine, Marc Denny explained that the name “Dog Brothers” originated from his observation that “*men* practicing the martial arts seemed to have the behavior patterns of a pack of dogs” (Pellitteri, 1998, p. 94; emphasis added). In turn, the gender balance at the Gatherings can be assessed by examining the lists of registered fighters that are published on the registration pages of recent or upcoming Gatherings (DBMA, 2023a,c). In 2023, the two U.S. Gatherings had 63 and 38 registered fighters, respectively. Judging by the names of the fighters, there were four female participants in each of the events.

Besides signaling that a Dog Brother is male by default, the official and unofficial communications of the Dog Brothers construct a certain kind of masculine ideal. As illustrated by the passage from Marc Denny quoted in Section 3.2, the men are tasked to “defend the land, women, and children of the tribe.” Furthermore, in addition to being male, a Dog Brother is also assumed to be heterosexual. Explaining the rationale behind the name, Marc Denny describes the Dog Brothers as follows: “[W]e are but a pack of dogs, and just like dogs we have territory, and hierarchy, and squabbles over the, females” (Guro Crafty, 2009). Being men, the Dog Brothers are also aggressive by nature and need an outlet for their aggression: ‘Aggression is an instinct, even as sex is an instinct. And just as a man eventually will have a nocturnal emission in the absence of sex, so too aggression will discharge eventually even in the absence of “legitimate” cause’ (Guro Crafty, 2009).

Recognizing the role of gender contributes to a deeper understanding of the ritual dynamics of the Dog Brothers Gathering of the Pack. The Gathering is an initiation rite, but more importantly, it is a *male* initiation rite.

Mikkelsen and Søgaard (2015) have analyzed male initiation rituals that involve a display of aggression that is carefully guarded from bursting into open hostilities. According to them, such rituals aim at maintaining the hegemonic masculine status quo in society. By demonstrating their potential for violence, men can claim superiority over women and non-hegemonic masculinities. However, by restricting the aggression to a ritual context, the men can stop it from escalating into overt intragroup conflict that would break homosocial bonds and thus ultimately threaten male dominance in the community. The dynamic is encapsulated well in a passage written by Marc Denny himself. According to Denny, men need to find outlets for their violent tendencies, lest “the discharge of the aggression becomes less predictable and often more dangerous” (Guro Crafty, 2009). For him, “[t]he solution is to ground aggression in a ritual expression that also prepares it for functional application.”

Mikkelsen and Søgaard’s (2015) own data is from the Bugkalot people of the Philippines, but they argue that similar dynamics can be observed in the industrialized West. More specifically, the men are caught in a peculiar double bind: they are expected to both use violence (or be feminized) and abstain from it (or be socially shunned). A solution is provided by real-contact stick fighting and other practices that allow for displays of aggression in a clearly bounded environment. Despite fighting with no holds barred, the Dog Brothers carefully keep the violence from spilling over into their relationships outside of combat. As already noted, the one rule that they are expected to obey is that they are friends at the end of the day.

## 4 Discussion

### 4.1 Studying extreme sport as a ritual

Extreme leisure sport challenges the perception of people as having a fundamental need for safety. If one of our primary aims as human beings is to minimize threats and harm to our well-being, why do some of us willingly climb steep rocks without the safety of a rope or, as in the case of the people discussed in this article, hit each other hard with a stick?

In this article, I suggest that at least some extreme sports utilize techniques that are also used in religious contexts to induce specific kinds of identity processes. As a case in point, I use “Gatherings of the Pack” organized by the stick-fighting group Dog Brothers. The gatherings involve common elements that in effect make them into rites of initiation that produce a sense of communitas (cf. Turner, 1977) and identity fusion (Whitehouse, 2018, 2021, pp. 93–99) among their participants. Noteworthily, the communitas is manifestly gendered; the participants are made into a community that is very specifically male.

I am definitely not the first to suggest that there are similarities between ritual and sport activities (for an overview, see Grimes, 2013, pp. 213–216). Especially worth noting is the work of Varda Burstyn who has argued that contemporary sports, and especially football, are in essence “male initiation rituals” that generate “the ideology of [− −] hypermasculinity, that is, an exaggerated ideal of manhood linked mythically and practically to the role of the warrior” (Burstyn, 2000, pp. 4 and 64). However, in contrast to previous works on the topic, I am focusing on a very specific kind of sport (extreme sports) and a very specific kind of ritual (imagistic rituals). I argue that the cognitive research on imagistic rituals adds novel understanding to the attractiveness of the danger inherent in certain kinds of extreme sport.

The concepts of imagistic ritual and identity fusion have been used in the martial arts context by Kavanagh et al. (2019). However, their focus is restricted to a specific promotion ceremony that is practiced in the context of Brazilian Jiu-Jitsu, namely, whipping the promoted student with belts. In this article, I argue that the notion of imagistic rituals has a more general applicability in the study of martial arts and extreme sports. In addition to promotion ceremonies and other practices that are rituals in the everyday sense of the word, extreme sports training itself may activate cognitive processes that are typical of religious rituals.

### 4.2 Identity fusion with an imagined community

What about extreme sports that are not group activities? Does my hypothesis of extreme sport as an imagistic ritual apply, for example, to free-solo climbing—a sport that is by definition practiced alone? I would argue that it does, for the simple reason that even individual sports are almost always practiced in a community. For example, there are very few people who devote themselves to a sport that is self-invented and has no other practitioners, just like there are very few people whose religious life consists of self-invented rituals. Even when the actual physical feat is performed alone (as in free-solo climbing), it is very often shared with a broader community, for example on social media (see Kidder, 2017, p. 51). In a sense, free-solo climbing is not too different from the vision quest practiced in many Native American communities (on the vision quest, see Tinker, 2005). Both involve withdrawing to solitude, enduring physical hardship, and returning to the community—or in ritual studies terminology, the three essential phases of a rite of passage: separation, liminal, and reincorporation (Van Gennep, 1960).

*It is a common theme in the professional literature to see the vision quest as definitive evidence of the radical individualism of Plains Native people. Nothing could be further from reality; in fact just the opposite is true. There is always a symbiotic relationship between the person engaging in the fast and the community to which she or he belongs, even though the ceremony involves the deeply personal sacrifice of rigorous fasting and prayer over several days. [- -] One quintessential sign of the communal nature of the vision quest is the common practice in Lakota and other communities for the people to greet the faster with a handshake and a thank-you for the faster’s accomplishment as she or he completes the ceremony. In a typical vision quest, moreover, the community or some part of the community participates in preparing the person to engage in the ceremony. There may be teachings that need to be shared and even preliminary ceremonies that have to be performed. Then through the duration of the fast the community will constantly be conscious of and in prayer for the one who is actually performing the ceremony in isolation* (Tinker, 2005, p. 9610).

*Mutatis mutandis*, everything in the excerpt above applies equally well to free-solo climbing. Despite being on the wall alone, a climber often trains and otherwise prepares for an ascent together with others. They may follow climbing media and, for example, read accounts that others have written about climbing the same rock. In all likelihood, they document their ascent with videos and photographs. By sharing these documents on social media, a climber can have their feat validated by the broader climbing community.

Investigating pilgrims to Santiago de Compostela, Lobato and Sainz (2020) found that the number of people they traveled with was not correlated with their experience of identity fusion. Accordingly, the community with which a pilgrim fuses may not be the one they have actually traveled with but, for example, the people with whom they have prepared for the pilgrimage (Lobato and Sainz, 2020, p. 504). Lobato and Sainz (2020) conclude that sharing the actual ritual experience with others does not appear to be necessary for the development of identity fusion. However, for the fusion to endure, it is important to have contact with other pilgrims and reflect on the experience with them *after* the pilgrimage.

Even if one always practices a sport alone, one can identify with an imagined community of co-athletes (e.g., “climbers”). Anderson (2016, p. 6) used the term “imagined community” about nation state and other groups with which people identify without ever meeting most of their members in person. However, I would argue that all groups are imagined in the sense that people always identify with a mental representation of the group (i.e., the prototype; Hogg, 2000, p. 226), not its flesh and blood members. Furthermore, and as noted by Swann et al. (2009, p. 995), it is possible to become fused with a group that is imagined in Anderson’s (2016) sense of the term. In a similar vein, Kapitány et al. (2020) and Ismer (2014) have suggested that watching televised events such as President Trump’s inauguration or the Football World Cup may serve as imagistic rituals that potentially facilitate identity fusion with the national collective or other imagined communities.

### 4.3 Why does anyone do this?

By now, I hope to have established a correspondence between the Dog Brothers Gathering of the Pack and imagistic rituals. However, the question that remains is: Why does anyone do them? What makes people attracted to Gatherings and practices in the imagistic mode in general? Whitehouse has discussed in depth how the two modes of religiosity support the efficient transmission and maintenance of a religious tradition. In other words, he focuses on why religious communities have adopted imagistic practices and not on why individual believers engage in them. Despite this, some preliminary hypotheses can be gleaned from the works of Whitehouse and others.

Cultural psychologists such as Shweder (1990) and Valsiner (2014, p. 1) have considered the search for meaning to be a, and perhaps the, key factor guiding human behavior and psychic functioning. Similarly, Heine et al. (2006) have proposed the so-called meaning maintenance model (MMM), the key idea of which is that a need for meaning is so fundamental that a lack of meaning in one area of life almost invariably leads to attempts to compensate for it elsewhere. The need for meaning may even be hardwired into the human brain; Bering (2002, p. 4, see also, 2003) has argued that humans are equipped with “Existential Theory of Mind”—“a biologically based, generic explanatory system that allows individuals to perceive meaning in certain life events.”

A sense of meaning is also a key factor driving identity work. In particular, the motivated identity construction theory (MICT; Vignoles et al., 2006) includes meaning in a list of identity motives. Having identities that fulfil identity motives evokes positive emotions. In contrast, unmet motives elicit negative emotions and often trigger a search for more satisfying identities.

In fact, and somewhat counterintuitively, extreme leisure sports and other high-risk activities that serve no obvious instrumental purpose may be particularly effective in facilitating personal meaning-making (cf. Whitehouse, 2021, p. 16). This is suggested, for example, by studies that show that when people do not receive concrete compensation from an activity, they attribute some other meaning to it. For instance, Liu and Sundar (2018; see also Festinger and Carlsmith, 1959) have demonstrated that when research participants are paid less for their participation, they perceive the participation to be more important and enjoyable. The explanation for this lies in cognitive dissonance: in order to maintain a consistent self-image, a person needs to perceive a correspondence between their motivations and activities. If an activity cannot be justified in terms of its concrete gains, some other meaning needs to be attributed to it. In turn, and according to Whitehouse (2021, p. 16), the defining feature of a ritual is that it has no causal link to any obvious goal. Therefore, activities that lack clear instrumental benefits may function similarly to rituals, providing a framework for individuals to derive personal meaning and cohesion.

As already noted, according to Whitehouse (2002), religious communities typically rely on doctrinal practices to transmit their official dogma. In turn, rituals in the imagistic mode are effective in inducing personal reflection and meaning-making. By activating episodic memory, imagistic rituals produce experiences that have a very personal quality. They become parts of one’s individual life story and consequently reveal something important about one’s own being. In addition, imagistic mode typically involves practices that are rarely performed and both sensually and emotionally arousing. They constitute a break in the everyday patterns of life and integrating them with one’s sense of self may therefore require some psychological work. In short, imagistic rituals serve personal meaning-making in ways that doctrinal rituals cannot. Because of this, imagistic rituals may be particularly effective in fostering identities that satisfy the fundamental human need for meaning.

## 5 Conclusion

In this article, I have reflected on the factors that attract people to leisure activities that pose a significant threat to their life and well-being. Of particular interest for me are activities in which the danger seems to have an intrinsic value. I focus on real-contact stick fighting as an example of such an activity. Stick fighting can be trained relatively safely by using protective equipment and banning the most dangerous techniques. However, the real-contact stick fighting practiced by the martial arts group Dog Brothers is marked by its almost complete lack of such safety protocols. Instead of being an unfortunate downside in an otherwise enjoyable activity, risk of death and injury is for the Dog Brothers a goal in itself. The sentiment is captured in the words of one of the original Dog Brothers and head instructor of the Dog Brothers Martial Arts, Marc “Crafty Dog” Denny, in a CNN interview (Conder, 2011): “We could make this a padded pillow fight but then it would lose its meaning. The danger and risk are necessary to the transformative nature of the experience.”

In this article, I strive to understand this (at least potentially fatal) attraction. On the basis of a ritual studies perspective, I make the following propositions:

1. The danger and hardship involved in an extreme sport activity is positively associated with identification with the extreme sport community. The “community” may be either a well-defined group of training partners (e.g., a climbing team), a fuzzy category of people practicing the same sport (e.g., “climbers” in general), or something in between.
2. The danger and hardship involved in an extreme sport activity is positively associated with identity fusion with the extreme sport community. In other words, the more danger is involved, the more a person’s personal identity overlaps with their social identity as an extreme sport athlete.
3. The danger and hardship involved in an extreme sport activity are positively associated with the sense of meaning gained from identifying with the extreme sport community.

In addition, I suggest that identities promoted in extreme sports and facilitated by imagistic rituals are manifestly gendered. Participation in an initiation ritual is very often restricted to those of a specific gender (Eliade, 2005, p. 4476). In particular, sport and ritual activities associated with men and masculinity are prone to utilize and perpetuate militant, “warrior” imageries. In addition, they are likely to involve ritualized performances of controlled aggression.

## Data availability statement

The original contributions presented in the study are included in the article/supplementary material, further inquiries can be directed to the corresponding author.

## Author contributions

TP: Writing – original draft.
